# The Utilization of Point of Care Ultrasound (POCUS) for the Confirmation of Gastric and Post-Pyloric Feeding Tube Placement in a Pediatric Intensive Care Unit

**DOI:** 10.24908/pocusj.v10i01.17785

**Published:** 2025-04-15

**Authors:** Alonso Marron, Michael S. Wolf, Marla Levine, Jeremy S. Boyd, Marta Hernanz-Schulman

**Affiliations:** 1 Division of Pediatric Critical Care, Vanderbilt University Medical Center, Nashville, TN, USA; 2 Division of Pediatric Emergency Medicine, Vanderbilt University Medical Center, Nashville, TN, USA; 3 Division of Emergency Medicine, Vanderbilt University Medical Center, Nashville, TN, USA; 4 Pediatric Radiology, Vanderbilt University Medical Center, Nashville, TN, USA

**Keywords:** pediatric, PICU, point of care ultrasound (POCUS), abdominal point of care ultrasound, enteral feeding tubes

## Abstract

The aim of this study was to investigate the role of point of care ultrasound (POCUS) as an alternative imaging modality to confirm the location of gastric and post-pyloric feeding tubes in patients admitted to the pediatric intensive care unit (PICU). This was a prospective descriptive study performed at a tertiary care children's hospital. Patients from birth to 17 years of age in whom the medical team placed a temporary enteral feeding tube were eligible for enrollment. The study physician, who was blinded to the radiographic findings, performed a POCUS study of the abdomen. An abdominal radiograph was obtained to confirm the placement in all patients. A total of 13 patients were enrolled, and 14 abdominal POCUS exams were completed. POCUS accurately identified the location of the enteral feeding tube in 10 of the 14 cases. POCUS had a sensitivity and specificity of 85.7% and 57.1%, respectively, in identifying gastric tubes. It had a sensitivity and specificity of 66.7% and 87.5%, respectively, in identifying post-pyloric tubes. No adverse events were reported. This study showed that POCUS had moderate sensitivity and specificity and was, overall, safe. Further studies can assess the level of training needed for improvement in accuracy, and larger studies can help support the findings of this data that POCUS is a safe and accurate alternative to radiographs for enteral feeding tube placement confirmation.

## Introduction

Enteral nutrition is associated with improved outcomes in critically ill children, including lower mortality, less organ dysfunction, and shorter hospital length of stay [1]. Malnourishment is associated with worse outcomes in patients admitted to the Pediatric Intensive Care Unit (PICU) [2,3]. Moreover, when compared to parenteral nutrition, enteral nutrition is associated with better outcomes in critically ill patients [1–5]. Enteral feeding tubes allow medical teams to safely provide adequate enteral nutrition to patients who are unable to eat by mouth. Moreover, enteral feeding tubes allow the administration of enteral medications that might not be available in other formulations. Given the known benefits of enteral nutrition and the utility of temporary enteral feeding tubes, feeding tubes are inserted frequently in hospitalized pediatric patients. One study reported the prevalence of temporary feeding tubes in pediatric hospitals to be 24% across the total neonatal and pediatric census of 63 participating pediatric hospitals with a prevalence of 18% in the PICU [6].

Despite the frequency with which temporary enteral feeding tubes are placed, current evidence demonstrates heterogeneity in the methods by which medical teams confirm the placement of enteral feeding tubes. Gastric aspirate pH, a common verification method, is validated in the literature, but the optimal pH used to confirm placement varies across studies [6–10]. Furthermore, this method cannot accurately distinguish between esophageal and gastric placement and obtaining a gastric aspirate sample immediately after the placement of a tube is not always possible [6–10]. Radiographs are considered the “gold standard” for verification of enteral tube placement, but this modality exposes patients to ionizing radiation. Additionally, if the feeding tube is not optimally placed or needs to be replaced, repeated radiographs may be required. One adult study showed that 33% of patients needing nasogastric tubes for nutrition or medication administration required three or more radiographs for enteral feeding tube placement during their hospital course [9]. Fluoroscopic guidance can also be utilized and can be considered the “gold standard” for placement of post-pyloric feeding tubes; nevertheless, this also exposes patients to ionizing radiation and is often infeasible due to lack of resource availability or clinical instability.

Point of care ultrasound (POCUS) represents a potential alternative imaging modality for bedside providers to verify the placement and location of enteral feeding tubes in patients admitted to the PICU. POCUS has the benefits of being able to be performed at the patient's bedside, is relatively inexpensive, has little to no risks for the patient, and does not expose patients to any form of ionizing radiation [11].

Several studies have demonstrated that POCUS can be used successfully for the placement and confirmation of nasogastric tubes and other enteral feeding tubes with comparable accuracy to radiographs [12–17]. Nonetheless, a Cochrane review of 10 studies assessing the accuracy of ultrasound for gastric tube placement found few studies with a low risk of bias, and concluded the evidence was too limited to state ultrasound had sufficient accuracy as a single test to confirm gastric tube placement [18]. Evidence is still lacking to determine the overall utility, safety, accuracy, and feasibility of using POCUS for this indication in children admitted to the PICU. Moreover, previous studies were performed in the pediatric emergency department and/or have had a pediatric radiologist or physician who is specialized in POCUS perform the ultrasound studies [12, 13]. Few studies have been done in the PICU with pediatric critical care physicians performing POCUS for this indication. Furthermore, few studies have assessed the accuracy of POCUS to determine the location of enteral feeding tubes placed in both gastric and post-pyloric locations in the pediatric population.

This study aimed to assess the accuracy and safety of utilizing POCUS to verify the placement and location of gastric and post-pyloric feeding tubes in patients admitted to the PICU with radiographs serving as the confirmatory gold standard. Our hypothesis was that POCUS is an accurate, feasible, and safe alternative imaging modality for the verification of placement and location of enteral feeding tubes in patients admitted to the PICU.

## Methods

### Enrollment/General

We conducted a prospective descriptive study from June 2023 to June 2024 at a single center in the PICU of a tertiary care children's hospital. This study was reviewed and approved by the institutional review board at Vanderbilt University Medical Center (IRB Number: 230253). All pediatric patients aged birth to 17 years admitted to the PICU who were determined by the primary medical team to require a nasally or orally inserted enteral feeding tube met inclusion criteria. Excluded from the study included patients with a history of abdominal/gastrointestinal surgery that alters the normal anatomy of the esophagus, stomach, and/or intestines (e.g., gastrostomy, bowel resection); patients with congenital abnormalities that alter the anatomy of the esophagus, stomach, and/or intestines. (e.g., duodenal atresia, omphaloceles, tracheo-esophageal fistulas); patients with presence of critical airway and/or current admission for airway reconstruction surgery; patients 18 years of age or older; patients with presence of c-collar or some other contraindication to neck ultrasound; patients whose primary language other than English or Spanish; and cases of parental refusal and/or patient refusal if developmentally able to assent. Patients were enrolled when the study physician was present, which was Monday through Friday from 1000 to 1600 hours. All patients were prospectively identified based on confirmation with the primary medical team of the requirement of enteral feeding tubes. Enrollment and exclusion criteria were assessed for each patient via the electronic medical record and communication with a parent and/or guardian at the bedside. Parents and/or legal guardians were asked to provide informed consent and patients 4 years and older were asked to provide assent for study participation if clinically and developmentally able. If a patient who had already been enrolled needed a new enteral feeding tube placed, the parent and/or guardian was approached and asked if they would like the patient to be re-enrolled. In these situations, the previously signed informed consent was utilized, and parents and/or guardians were asked to re-confirm enrollment.

### Protocol

Nasal and oral enteral feeding tubes were placed by the bedside nurse per unit policy. An abdominal radiograph was obtained on all patients to confirm the location of the enteral feeding tube per unit practice. The decision to place a gastric or post-pyloric feeding tube was made by the primary medical team with no input from the study physician or study team. The study physician performed an abdominal POCUS examination on each patient after the bedside nurse placed the enteral feeding tube. The study physician was blinded to the results and images of the abdominal radiograph at the time of the POCUS study. A maximum of 5 minutes was allotted for each abdominal POCUS. The protocol stated that if the study physician could not visualize the enteral feeding tube during the abdominal study, transverse images of the neck would be obtained with POCUS to evaluate for placement of the tube in the cervical esophagus. The maximum time allotted to obtain the neck images was two minutes. All images and video clips were saved in the encrypted, Health Insurance Portability and Accountability Act (HIPPA)-compliant database QPATH (Telexy, Blaine, Washington). The study physician then filled out a survey with his interpretation of the POCUS study as well as other demographic and relevant information about the study such as the length of time the study took and whether any complications were encountered. Each patient enrolled was assigned a de-identified study name and number combination; this combination was used to label the patient's images and video clips for future reference and data collection. A pediatric radiologist then assessed the images and video clips for each study and filled out a separate survey with her interpretation for quality assessment. All results from the surveys were stored in encrypted databases to which only the study team had access. POCUS study interpretation and demographic data were documented and saved in a data collection form stored in an encrypted software to which only the study team had access. The interpretation of the study physician was then compared to the final interpretation of the abdominal radiograph.

### Physician Training

The study physician was a pediatric critical care fellow who underwent education about obtaining gastric and pyloric/post-pyloric images to determine enteral feeding tube placement and subsequently served as the primary sonographer for the study. The training involved a 1-hour hands-on session instructed by a pediatric radiologist utilizing 2 patients in the PICU who had enteral feeding tubes in place. This session involved correct technique and correct anatomic identification. The study physician additionally completed the Society of Critical Care Medicine Critical Care Ultrasound: Pediatric and Neonatal Course.

### Imaging Technique

**Figure 1.**
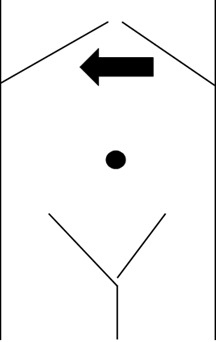
Diagram of the transducer placement on the epigastrium. The solid arrow represents the transducer with the arrow pointing to the location of the transducer marker. The transducer was fanned inferiorly across the epigastrium for visualization of stomach, visualizing the fundus, body, and – subsequently – the antrum.

**Figure 2.**
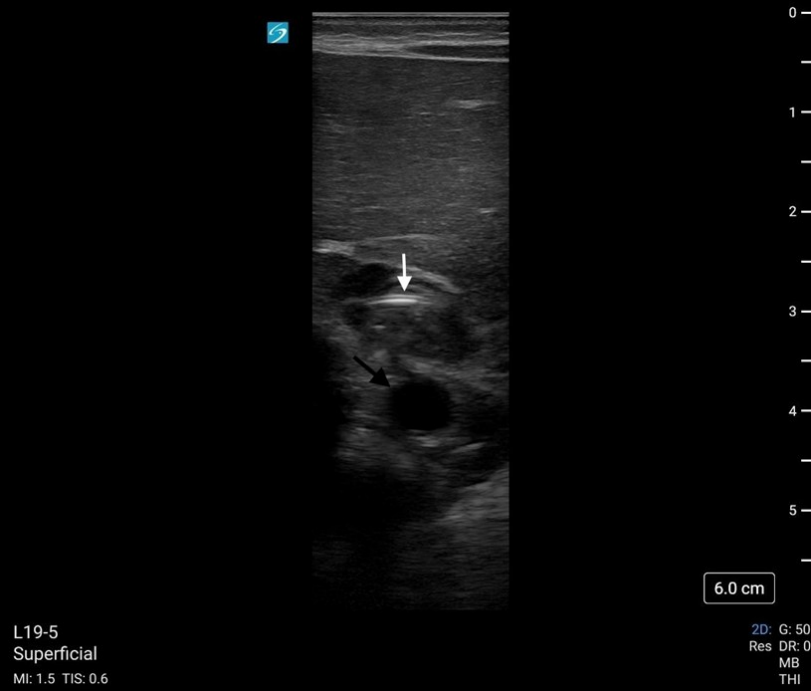
Enteral feeding tube (white arrow) visualized in esophagus using point of care ultrasound (POCUS). The aorta (black arrow) can be seen posterior to the esophagus and is used as a landmark.

**Figure 3.**
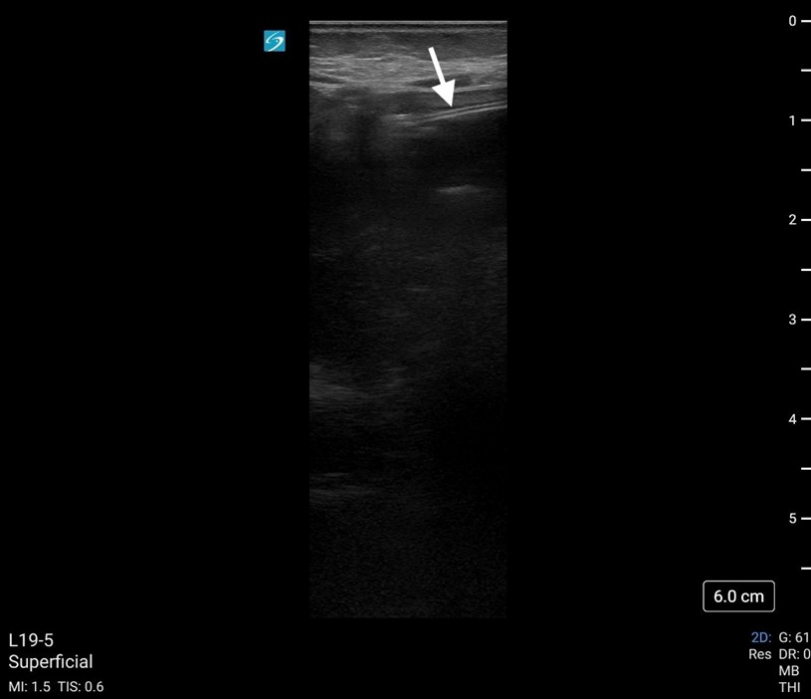
Enteral feeding tube (white arrow) demonstrated in antrum of stomach using point of care ultrasound (POCUS). Feeding tube visualized as 2 parallel hyperechoic lines.

**Figure 4.**
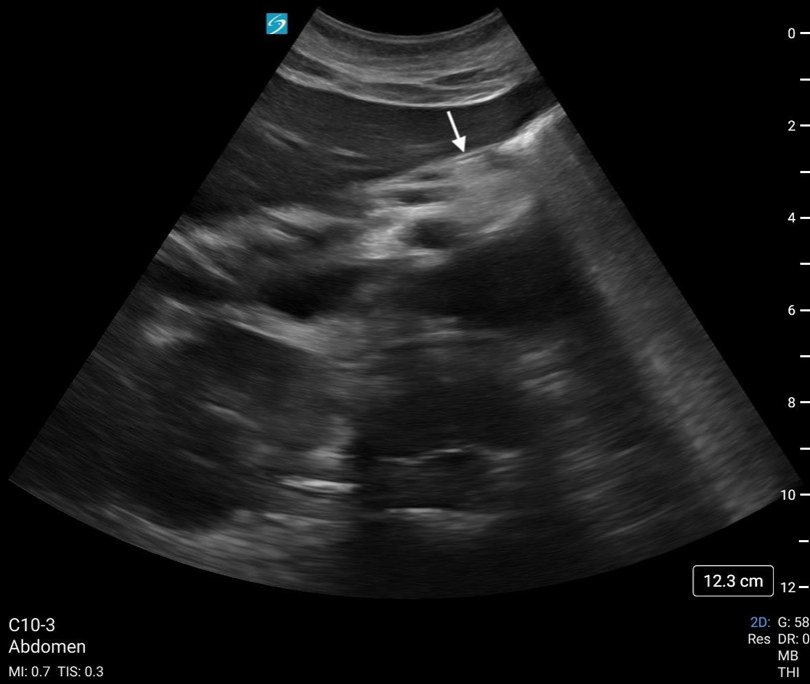
Enteral feeding tube (white arrow) visualized in the immediate post-pyloric region using point of care ultrasound (POCUS)

Images were obtained by the study physician using a Sonosite PX Ultrasound Machine (Fujifilm, Bothell, Washington). A curvilinear transducer with a frequency of 10-3MHz (C10-3) and a linear transducer with a frequency of 19-5MHz (L19-5) were used for the abdominal studies, depending on patient size and field of view. The transducer was placed over the patient's abdomen while the patient was supine (Figure 1). The transducer was placed in the transverse position in the subxiphoid area to identify the aorta and the esophagus at the gastroesophageal junction (shown in Figure 2) and then moved caudally while maintaining a transverse orientation following the stomach from fundus to antrum until the duodenum was reached near the head of the pancreas. The enteral feeding tube appeared as two hyperechoic parallel lines (Figure 2 and Figure 3). Images and video clips were taken of these areas regardless of whether the study physician visualized the enteral feeding tube. The study physician would then move the transducer along the antrum of the stomach and attempt to visualize the pyloric and post-pyloric regions. The duodenum around the head of the pancreas was used as the endpoint. A slight counterclockwise rotation from the transverse orientation of the transducer was utilized as needed to better visualize the post-pyloric region. Landmarks such as the medial and posterior neck of the gallbladder, the abdominal aorta, the inferior vena cava, the superior mesenteric artery, and the superior mesenteric vein were used to assist with the location of the first parts of the duodenum. Figure 4 demonstrates an example of a post-pyloric feeding tube visualized with POCUS. Images and video clips of this area were taken regardless of the visualization of the enteral feeding tube. If the enteral feeding tube was not visualized in the allotted 5 minutes, the protocol included obtaining images of the neck to assess the esophagus and airway with a linear transducer (L19-5MHz). The transducer was to be placed on the lateral and/or anterior aspect of the patient's neck in the transverse position. The enteral feeding tube appeared as a hyperechogenic ring or a double line, often demonstrating posterior acoustic shadowing, depending on the tube's orientation with respect to the transducer [12]. Longitudinal scans were also performed if unable to visualize the enteral feeding tube. Images were taken of these areas regardless of whether the performing physician visualized the feeding tube or not. The maximum amount of time for this portion of the exam was 2 minutes. Abdominal radiographs were obtained on all patients, and the radiographs were interpreted by a pediatric radiologist as part of routine care.

### Data Collection

Data obtained from the electronic medical records included age, height, weight, body mass index, sex, race/ethnicity, primary diagnosis, chronic medical diagnoses, level of respiratory support, sedation/analgesic infusions being administered, intermittent sedation/analgesic medication the patient was receiving/had received, study physician assessment of enteral feeding tube location, time taken to complete POCUS study for both abdominal and neck studies, and radiologist final report of the abdominal radiograph of the location of the enteral feeding tube.

### Data Analysis

The accuracy of POCUS to determine the location of enteral feeding tubes was calculated. Specificity and sensitivity were calculated for gastric and post-pyloric feeding tubes utilizing abdominal radiographs as the standard. For the sensitivity and specificity of utilizing POCUS to identify gastric feeding tubes, true positive results were the studies in which both POCUS and the radiographic report determined the feeding tube was in the stomach. False positive results were the studies in which POCUS determined the feeding tube was in the stomach, but the radiographic report reported the tube was in another location (i.e. post-pyloric region). False negative results were the studies in which POCUS determined the feeding tube was not in the stomach (i.e. post-pyloric or not visualized), but the radiographic report reported the feeding tube was in the stomach. True negative studies were the studies in which both POCUS and the radiographic report determined the tube was not in the stomach. The sensitivity and specificity of utilizing POCUS to identify post-pyloric feeding tubes were calculated in a similar method. The 95% confidence intervals were calculated utilizing Clopper Pearson confidence limits. Means and medians were also calculated when appropriate.

## Results

A total of 21 patients were approached for enrollment. Two patients had families that declined participation in the study and one had a parent that provided informed consent, but the patient declined to provide assent. One patient was initially enrolled, and the parent provided informed consent, but upon further history gathering from the parent, the patient met exclusion criteria, and no POCUS exam was performed on this patient. With this, 17 patients were enrolled in the study. Two patients were part of a teaching session with the pediatric radiologist to learn the technique at the beginning of the study. These images were not utilized for final data analyses and neither patient required replacement of a temporary feeding tube. One patient never had an enteral feeding tube placed. One patient required interventional radiology for placement of the enteral feeding tube and never had a POCUS study done. Of the remaining 13 patients, 1 patient had 2 POCUS studies performed. Therefore, a total of 14 POCUS examinations were included in the study. Table 1 demonstrates all POCUS studies with demographic information, enteral feeding tube location determined by POCUS, and enteral feeding tube location determined by the abdominal radiograph. No neck POCUS studies were needed to be performed. The age of the patients ranged from 19 days to 15 years of age with a median of 84 days and a mean of 1372.1 days of age or 3.8 years of age (SD=2103 days or 5.8 years). A total of six boys and seven girls (53.8%) were enrolled in the study. The median time taken for the abdominal POCUS studies was 249 seconds and the mean was 244.5 seconds (SD=49.9 seconds). Demographic information and characteristics of the enrolled patients can be found in Table 1.

**Table 1.** Point of Care Ultrasound (POCUS) Study Numbers listed with Demographic Information and Results

Study Number	Age	Primary Diagnosis	Respiratory Support	Location of Feeding tube per Study Physician	Time Taken to Complete POCUS Study (min:seconds)	Location of Feeding Tube per abdominal Radiograph
1	3 weeks-old	Apnea	Intubated/Mechanical Ventilation	Gastric	05:00	Gastric
2	15 years-old	Acute Respiratory Failure with hypoxia	Intubated/Mechanical Ventilation	Gastric	03:18	Feeding tube folded in mid-esophagus; Replogle in stomach
3	4 months-old	Acute Hypoxic Respiratory Failure	High Flow Nasal Cannula	Gastric	04:15	Gastric
4	4 months-old	Acute Hypoxic Respiratory Failure	High Flow Nasal Cannula	Gastric	03:40	Post-Pyloric
5	7 years-old	Fulminant Liver Failure	Intubated/Mechanical Ventilation	Gastric	04:03	Peri-Pyloric/antrum of Stomach
6	2 months-old	RSV Bronchiolitis	NIPPV, BiPAP	Gastric	05:00	Level of the Pylorus/Stomach
7	2 months-old	Acute Hypoxic Respiratory Failure	NIPPV, BiPAP	Post-Pyloric	04:15	Proximal duodenum
8	19 days-old	Acute Hypoxic Respiratory Failure	High Flow Nasal Cannula	Post-Pyloric	03:25	Proximal duodenum
9	2 months-old	Bronchiolitis	High Flow Nasal Cannula	Post-Pyloric	03:55	Post-pyloric
10	27 days-old	Acute Bronchiolitis due to RSV	High Flow Nasal Cannula	Gastric	04:55	No feeding tube visualized; Tube was coiled in oropharynx
11	11 months-old	Acute Bronchiolitis due to RSV	High Flow Nasal Cannula	Gastric	05:00	Just beyond pylorus
12	43 days-old	RSV Bronchiolitis	High Flow Nasal Cannula	Gastric	04:01	Gastric
13	9 years-old	Acute Respiratory Failure	Intubated/Mechanical Ventilation and ECMO	Post-Pyloric	04:17	Gastric
14	13 years-old	Rhabdomyosarcoma	Intubated/Mechanical Ventilation	Post-Pyloric	01:59	Duodenal Bulb

The study physician appropriately identified the location of the enteral feeding tubes in 10 of the 14 studies (71.4%). For determining the location of enteral feeding tubes located in the stomach, POCUS had a sensitivity of 85.7% (CI 42.1-99.6%) and a specificity of 57.1% (CI 18.4-90.1%). For determining the location of post-pyloric feeding tubes, POCUS had a sensitivity of 66.7% (CI 22.3-95.7%) and a specificity of 87.5% (CI 47.4-99.7%). No adverse events were reported during any of the studies. Patient movement and excessive air in the gastrointestinal tract were reported as the biggest difficulties the study physician had during the performance and interpretation of the images and videos. All images and videos were assessed by a pediatric radiologist for quality assurance who determined the images and videos to be adequate.

**Figure 5.**
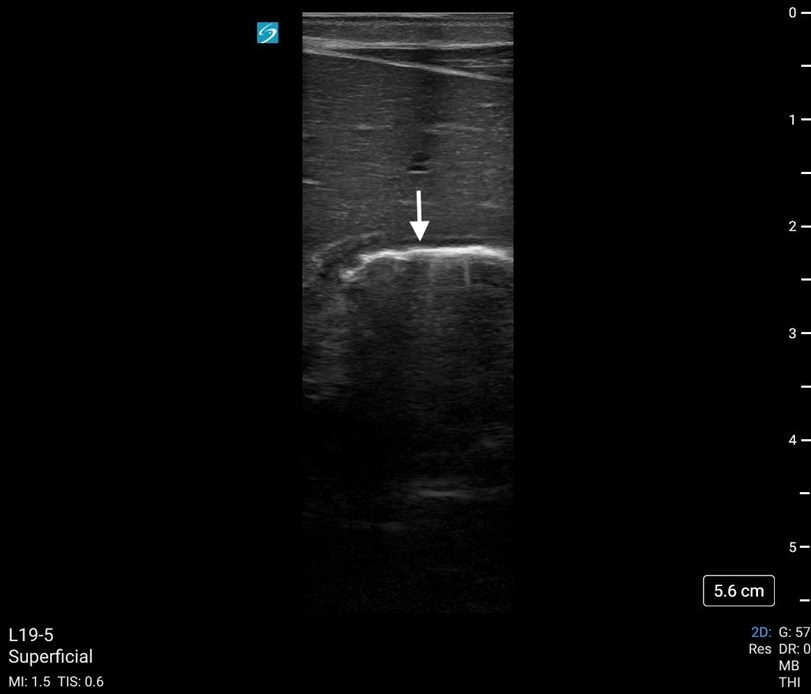
Gastric mucosa (white arrow) can be seen as a hyper-echoic structure. Reverberation artifacts and reported gastric and intestinal distention can lead to false-positive point of care ultrasound (POCUS) finding.

One false positive was reported. The study physician had recorded the enteral feeding tube as being in the stomach. After reassessment with the bedside nurse and the radiographic imaging, it was determined that the enteral feeding tube had coiled in the oropharynx. The quality assurance pediatric radiologist was not able to visualize a tube in any of the images or videos acquired during the POCUS study (Figure 5). The error appeared to be related to artifacts produced by bowel gas.

One case reported the enteral feeding tube as being in the stomach. Radiographic images and POCUS images were assessed by the study team, and a tube was indeed present in the stomach in the POCUS images and the abdominal radiograph. This tube, however, was a gastric decompressive tube, and the enteral feeding tube was folded in the mid-esophagus. Since POCUS is unable to differentiate between tube types, the study team determined this was a positive examination given that the study physician had accurately visualized the tube in the stomach with POCUS.

Of the studies performed, seven were on patients on high flow nasal cannula and five were on patients that were intubated on mechanical ventilation. One of these five patients intubated on mechanical ventilation were also on V-V ECMO, and two were on patients on noninvasive positive pressure ventilation (NIPPV). POCUS accurately determined the location of the enteral feeding tube in four of the seven studies (57.1%) done on patients on high flow nasal cannula; two out of the two studies (100%) done on patients on NIPPV and four out of the five studies (80%) done on patients intubated on mechanical ventilation. Five out of the 14 studies (35.7%) were done on patients receiving a sedation and/or analgesic infusion with four out of five (80%) of these studies accurately determining the location of the feeding tube, compared to only six out of nine (66.7%) in patients receiving no sedation and/or analgesic infusions. Table 2 shows the results of the studies by different demographic characteristics and sorted by visualization of the enteral feeding tube. Table 3 shows the results of the studies by different demographic characteristics and by whether POCUS accurately visualized the location of the enteral feeding tube.

**Table 2.** Demographic and Characteristics of Patients

Total N=13		
**Sex**		
Male	6	46.2%
Female	7	53.8%
**Age**		
Neonate (0-1 months)	3	23.1%
Infant (1 month-1 year)	6	46.1%
Toddler (2-5 years)	0	0
School-Age (6-12 years)	2	15.4%
Adolescent (13-<18 years)	2	15.4%
**Weight, Kg (Total N=13)**		
Mean (SD)	19.5 (23.7)	
Range	2.97-74.4	
**Height, cm (Total N=10)**		
Mean (SD)	89.45 (48.7)	
Range	21.3-164.3	
**Race/Ethnicity**		
White	8	61.5%
Hispanic or Latino/a	3	23.1%
Black or African American	2	15.4%
**Primary Diagnosis**		
Acute Respiratory Failure/Apnea	6	46.2%
Viral Bronchiolitis	5	38.5%
Liver Failure	1	7.7%
Oncologic Disease	1	7.7%
**Chronic Medical Diagnosis/-es**		
None	6	
Prematurity	4	
Asthma	2	
Developmental Delay	1	
Seizure Disorder	1	
Cerebral Palsy	1	
Oncologic Disease	1	
Pyloric Stenosis	1	
Cardiac Disease	1	
Obesity	1	
**Analgesic/Sedation infusion**		
None	8	61.5%
Dexmedetomidine	3	23.1%
Dexmedetomidine and Opioid (hydromorphone, fentanyl)	2	15.4%
**Respiratory support (Total N=13)**		
High Flow	6	46.1%
NIPPV	2	15.4%
Intubated/Mechanical Ventilation	5	38.5%
**Enteral Feeding tube location based on radiographic report (Total N=14)**		
Stomach	6	42.8%
Post-pyloric	6	42.8%
Esophagus	1	7.14%
Not visualized	1	7.14%

**Table 3.** Demographics and Characteristics by Visualization of Gastric and Post-pyloric Feeding Tubes

	No (N=4)	Yes (N=10)	Total (N=14)
**Sex**			
Male	2 (28.6%)	5 (71.4%)	
Female	2 (28.6%)	5 (71.4%)	
**Time, seconds**			
Mean	268 (SD=37.3)	235.1 (SD=52.8)	
Range	220-300	119-300	
**Age range**			
Neonate (0-1 months)	1	2	
Infant (1 month-1 year)	2	5	
Toddler (2-5 years)	0	0	
School-Age (6-12 years)	1	1	
Adolescent (13-<18 years)	0	2	
**Feeding tube Location per radiograph**			
Gastric (N=6)	1 (20%)	5 (80%)	
Post-pyloric (N=6)	2 (33.3%)	4 (66.7%)	
Esophagus (N=1)	0	1 (100%)	Counted as positive. Gastric suction tube visualized on POCUS
Oropharynx (N=1)	1 (100%)	0	
**Respiratory Support**			
High Flow (N=7)	3	4	
NIPPV (N=2)	0	2	
Intubated/ Mechanical Ventilation (N=5)	1	4	
**Sedation/Analgesic Infusions**			
None	3	6	
Dexmedetomidine	0	3	
Dexmedetomidine and Opioid (Hydromorphone, Fentanyl)	1	1	

## Discussion

This prospective descriptive study demonstrated that using POCUS for the localization of gastric or post-pyloric feeding tubes in patients in the PICU is a safe and rapid procedure. However, it has variable accuracy potentially dependent on the experience of the operator and the patient population. To our knowledge, this is the first study that could be found in the literature that set out to determine the accuracy of POCUS to confirm the placement of both gastric and post-pyloric feeding tubes in the PICU setting. While some previous studies assessing gastric tubes have demonstrated higher sensitivity, they involved investigators with advanced training in performing and interpreting abdominal ultrasound images [12, 13, 17]. This study involved a single user who did not have extensive fellowship training in POCUS. The accuracy of POCUS is highly dependent on the sonographer's technique and ability to interpret the images. Furthermore, multiple patient-dependent factors affect the accuracy of POCUS for this indication. The patient's body habitus and the presence of gastrointestinal gas, either in the stomach which can surround the enteral feeding tube, or in other bowel loops, such as a distended gas-filled colon, affect the ability to identify the enteral tubes and surrounding structures. For example, in this study, 10 of the examinations were performed on children less than 12-months of age. Many of the patients would cry or move around during the POCUS study, making it difficult to visualize structures or follow structures once visualized. Other reports have described similar challenges. The limited scanning experience of the study physician likely contributed to the difficulty of bypassing obstacles related to overlying bowel gas and patient movement and suggests that added experience would likely correlate with an increase in accuracy.

One study done in a pediatric emergency department found POCUS to have a sensitivity of 88% for identifying naso- or oro-gastric tubes [13]. The ultrasound images were interpreted by an ultrasound-trained pediatric emergency department physician and focused only on gastric tubes. Another study reported ultrasound had a sensitivity of 100% in identifying temporary gastric tubes in the PICU; the study only assessed gastric tubes, and a radiologist performed the bedside ultrasound and interpreted the images [12]. More studies should focus on assessing the amount of training and practice pediatric critical care providers require to be able to utilize POCUS to attain the highest accuracy for enteral feeding tube placement confirmation. Other factors that proved challenging and could have influenced the relatively low sensitivity of this study include the amount of POCUS studies done on patients on high-flow nasal cannula. This method of respiratory support potentially increases the amount of aerophagia and gastric distention in a patient. This can lead to a gas-filled gastrointestinal tract which contributes to reverberation and artifacts on ultrasound images and obscures the tube from the ultrasound beam. Further evaluations can focus on assessing the accuracy of POCUS for enteral feeding tube location in different types of respiratory support in pediatric patients.

Despite the findings of this small study, POCUS could still prove an accurate imaging modality for the placement and assessment of enteral feeding tubes in the pediatric population. A study in 2021 demonstrated that ultrasound-guided post-pyloric feeding tube placement in patients admitted to the PICU had a higher first-pass success rate compared to blind placement (94% vs 57%) with no adverse events or complications [15]. The median insertion time for an ultrasound-guided post-pyloric tube was 18 minutes [15]. Hamadah et al. published a similar study in 2021 on infants in the pediatric cardiac intensive care unit. They showed a first-pass success rate of 87% with a median insertion time of 15 minutes, and no complications or adverse events [14]. Although these studies demonstrated ultrasound-guided post-pyloric feeding tubes are possible with a high success rate in pediatric patients admitted to the PICU, questions remain. These relate to the feasibility of this technique given the time commitment required for the procedure and the amount of training needed for bedside nursing and pediatric critical care providers to appropriately perform this procedure. At the time of this study, no studies had been done to exclusively assess the accuracy of POCUS utilized by a pediatric critical care physician to determine the location of gastric and post-pyloric enteral feeding tubes.

In this study, one of the POCUS examinations correctly demonstrated a tube in the stomach. As stated before, this was determined to be a gastric decompressive tube and not an enteral feeding tube. POCUS was unable to tell the type of tube visualized or distinguish between more than one tube in the gastrointestinal tract. This could limit the ability of providers to utilize POCUS for this indication in patients with a gastric suction tube that cannot be temporarily removed. POCUS, nonetheless, is a feasible and accurate method of confirming nasogastric tubes and oral gastric tube placement in pediatric patients presenting to the pediatric emergency department [12].

There were several limitations to this study. The small number of cases limited the ability to make strong conclusions about the accuracy of POCUS in determining the location of gastric and post-pyloric feeding tubes in patients in the PICU. Moreover, no enteral feeding tubes were placed in the trachea or airway, thus, the ability of POCUS to identify enteral feeding tubes incorrectly placed in the airway and/or lungs could not be assessed. Additionally, pediatric patients are widely heterogeneous populations regarding age and size. This necessitates the use of different techniques and transducers for optimal imaging depending on these and other factors. In this study, 4 patients were above the age of 12 months while the rest of the 10 POCUS studies were performed on patients less than 1 year of age. This limits the ability to make generalized conclusions about the accuracy of POCUS in older pediatric patients for this indication. More studies, therefore, should be done to further assess the accuracy of POCUS for enteral feeding tube placement confirmation in different pediatric age ranges. Another limitation is the lack of any neck POCUS studies performed. One adult study demonstrated the high sensitivity of ultrasound for gastric tube placement in adult patients in the intensive care unit by performing a neck POCUS study and then an epigastric POCUS study [19]. Given that the protocol for this study only had neck POCUS studies performed in cases where the study physician could not visualize the feeding tube in the abdominal ultrasound images, the exclusion of the collection of ultrasound images of the neck on the patients in this study could have affected the sensitivity and specificity of POCUS.

## Conclusion

At the time of this study, no known studies had been done to exclusively assess the accuracy of POCUS utilized by a pediatric critical care physician to determine the location of gastric and post-pyloric enteral feeding tubes. POCUS for confirmation of gastric and post-pyloric feeding tubes in pediatric patients admitted to the PICU represents a promising imaging modality. Future studies should evaluate the optimal level of training, the best protocol for this indication, and the optimal patient population for this indication with respect to age, respiratory support, and gaseous distention. More studies could encourage clinicians to invest in POCUS training for this method as it could help reduce radiation exposure in pediatric patients and represent a more time-effective modality for enteral feeding tube location verification when compared to radiographs. Analyses of the costs and resource utilization associated with POCUS and radiographs, when utilized for the confirmation of enteral feeding tube placement, might also be explored in future studies. This study and future studies will help realize the potential of POCUS as an adequate, feasible, safe, and better alternative to radiographs in the confirmation of enteral feeding tubes. Thus, helping reduce the number of X-rays in children admitted to the PICU and reducing the exposure to ionizing radiation in this population.






